# Angiopoietin-like 3 (ANGPTL3) drives cell proliferation, migration and angiogenesis in cervical cancer via binding to integrin alpha v beta 3

**DOI:** 10.1080/21655979.2021.2024951

**Published:** 2022-01-18

**Authors:** Lijun Zhong, Lin Tang, Xiaoxia He

**Affiliations:** Department of Gynecology, The Affiliated Hospital of Traditional Chinese Medicine, Southwest Medical University, Luzhou, China

**Keywords:** ANGPTL3, alpha v beta 3 (αvβ3), angiogenesis, cervical cancer, integrin, migration

## Abstract

Angiopoietin-like 3 (ANGPTL3) has been uncovered to play an oncogenic role in several kinds of human malignancies. Nevertheless, whether ANGPTL3 functions in cervical cancer (CC) has not yet been reported. This paper is intended to explore the impact of ANGPTL3 on CC cells and elucidate the potential mechanism. In this study, quantitative real-time polymerase chain reaction (qRT-PCR) and Western blot were performed to analyze the ANGPTL3 expression. Western blot was also performed to examine integrin αvβ3 protein level. Cell proliferation was evaluated by MTT assay, EdU staining and Western blot analysis. In addition, the migratory and invasive abilities of cells were, respectively, estimated by wound healing and transwell assays. Tube formation assay was performed to determine endothelial cell angiogenesis. Levels of vascular endothelial growth factor (VEGF) and vascular endothelial growth factor receptor 2 (VEGFR2) were measured by ELISA. As a result, ANGPTL3 expression was significantly higher in CC cells relative to that in normal cervical cells. Silencing of ANGPTL3 suppressed cell proliferation, migration and invasion. Besides, downregulation of ANGPTL3 inhibited human umbilical vein endothelial cell (HUVEC) angiogenesis and repressed protein level of integrin alpha v beta 3 (αvβ3). Upregulation of αvβ3 offsets the inhibitory effect of ANGPTL3 on proliferation, migration and invasion in CC cells. Upregulated expression of αvβ3 promoted blood vessel formation and secretions of VEGF and VEGFR2. In conclusion, ANGPTL3 silencing may serve as a tumor suppressor in CC through integrin αvβ3, which provides a potentially novel therapeutic target for patients with CC.

## Introduction

Cervical cancer (CC) ranks the fourth most frequent cancer among women around the world and 10–15% of cancer deaths in women owing to the gynecological cancer [1,2]. Moreover, the 5-year survival rate of early CC is about 85%, while that of most patients with CC at advanced stages is no more than 40% [3,4]. High rates of recurrence and metastasis are responsible for poor clinical outcomes of CC patients and unsatisfactory treatment effectiveness [5]. Thus, it is essential to further understand the pathogenesis of CC and develop novel effective anti-metastasis drugs for the treatment of CC.

Angiopoietin-like protein 3 (ANGPTL3), a member of angiopoietin-like proteins (ANGPTLs), is a multifunctional secreted protein that is mainly expressed in the liver [6]. It is well documented that ANGPTL3 participates in multiple biological processes, such as angiogenesis and lipid metabolism [7,8]. Recent studies have found that ANGPTL3 also plays a vital role in the occurrence and development of cancers [9–11]. For example, ANGPTL3 displayed elevated expression in esophageal cancer tissues [12]. Circulating ANGPTL3 and ANGPTL4 expression was obviously elevated in patients with hepatocellular carcinoma (HCC) compared with patients with chronic hepatitis and the controls [13]. In addition, high expression level of ANGPTL3 was demonstrated to be relevant to shorter survival in patients with high-grade serous ovarian (HGSC) cancer [14].

The integrin family of cell adhesion molecules contributes to the attachment of cells to the extracellular matrix and cell–cell interactions [15]. Integrin alpha v beta 3 (αvβ3) is one of the most important cell surface receptors, combining with fibronectin, laminin, collagen and osteopontin, which are defined as extracellular matrix proteins [16,17]. Accumulating evidence has shown that αvβ3 plays a significant role in angiogenesis and metastasis in tumors [18]. It has been suggested that ANGPTL3 participates in the process of angiogenesis, adhesion and migration of endothelial cells via binding to αvβ3 [19]. However, whether the combination of ANGPTL3 and αvβ3 affects biological activities of cervical cancer cells is still uncertain.

In the present study, we hypothesized that ANGPTL3 may regulate cell proliferation, migration and angiogenesis in cervical cancer through binding to αvβ3. We first analyzed ANGPTL3 expression in various cervical cancer cell lines. Then, the effects of ANGPTL3 on cell proliferation, invasion and migration and angiogenesis in cervical cancer and αvβ3 expression were analyzed. Finally, the effects of αvβ3 on ANGPTL3 knockdown for cell biology were explored.

## Materials and methods

### Cell culture

Human normal cervical cell line Ect1 and CC cell lines HeLa, C-33A, SiHa, CaSki, and HCC-94 and human umbilical vein endothelial cells (HUVECs) were provided by Cell Bank of the Chinese Academy of Science (Shanghai, China) and kept in RPMI-1640 medium (Gibco, Grand Island, NY, USA), to which 10% fetal bovine serum (FBS, Gibco, Rockville, MD, USA) was added. The growth condition was 37°C with 5% CO_2_.

### Cell transfection

For the knockdown of ANGPTL3, the specific shRNA targeting ANGPTL3 (shRNA-ANGPTL3-1/2) and corresponding control shRNA (shRNA-NC) were obtained from Gene Pharma (Shanghai, China). To overexpress αvβ3, Integrin-αv-specific pcDNA overexpression vector (oe-Integrin-αv), Integrin-β3-specific pcDNA overexpression vector (oe-Integrin-β3) and corresponding control empty vector (oe-NC) were obtained from Gene Pharma. Lipofectamine 2000 reagent (Invitrogen, USA) was adopted for plasmid transfection in light of the manufacturer’s instructions. Eventually, the transfected cells were harvested at 48 h.

### MTT assay

MTT assay was performed to appraise cell proliferation. Briefly, transfected or untreated cells were seeded into 96-well plates. Each group was repeated six times. The medium in each well was removed, and the cells were added with 100 μg of MTT in each well at 24, 48, and 72 h of cultivation, respectively. The medium was discarded, followed by the addition of 100 μL dimethyl sulfoxide (DMSO) to dissolve the formazan crystals after incubation for another 4 h. The absorbance reading of each well was detected with a microplate reader (BioRad) at 490 nm.

### EdU staining

For EdU incorporation assay, HeLa cells were seeded into 96-well plates for 48 h at 37°C. Then, cells were followed 4% formaldehyde fixation for 15 min and 0.5% Triton X-100 permeabilization for 20 min at room temperature after the supplementation of 50 µM of EdU for another 4 h at 37°C. 100 µl of 1× Apollo staining reaction solution was used to incubate the cells for 30 min followed by Hoechst33342 (100 µl) staining for 30 min after washed in PBS thrice. Images were captured by a fluorescent microscope (Olympus BX53, Japan).

### Quantitative real-time polymerase chain reaction (qRT-PCR)

Total RNA from CC and control cells was extracted using the TRIzol kit (Invitrogen). The cDNA was synthesized using PrimeScript RT Master Mix (Takara, Japan). Amplification of the cDNA was performed by PCR reactions with the SYBR Premix Ex Taq™ II kit (Takara, Shiga, Japan). The relative expression of the target genes was calculated by relative quantification (2^−ΔΔCq^) method. All ANGPTL3 expression data were normalized to GAPDH from the same sample. Each experiment was carried out in triplicate.

### Wound healing assay

Transfected cells added to a six-well plate were inoculated in RPMI-1640 containing 10% FBS at 37°C until cell growth reached 80–90% confluency. Then, a white pipette tip was used to create a wound in the cell monolayer, followed by washing three times with phosphate-buffered saline. Cell migration into the wound surface in five randomly selected fields was observed under an inverted microscopy 24 h later.

### Transwell assay

For transwell invasion assay, transwell chambers (Corning Incorporated, Corning, NY) coated with 0.1 mL of matrigel (BD Biosciences, San Jose) were used. Untreated cells or transfected HeLa cells with or without treatment of oe-Integrin-αv/oe-Integrin-β3 were collected and suspended. Cell suspensions were then loaded into the upper compartment, and the lower chamber was loaded with a medium with 10% FBS. After incubation for 24 h, cells on the upper face of the transwell membrane were wiped off. 100% methanol was used to fix invaded cells, and hematoxylin and eosin were added for staining. A microscope was applied for cell counting in five random visual fields for each chamber. The experiment was independently performed in triplicate.

### Endothelial tube formation assay

Transfected cells were seeded into six-well plates and cultured in RPMI-1640 at 37°C with 5% CO_2_. After 24 h incubation, cell supernatant in each group was harvested. Transfected HUVECs were cultured in cell supernatant with or without treatment of oe-Integrin-αv/oe-Integrin-β3 and plated in 24-well plate with matrigel (BD Biosciences, San Jose) pre-coating. After 12 h incubation, tubules were quantified by microscopy and assessed by Image Pro Plus software. Three independent experiments were required for each treatment.

### Enzyme-linked immunosorbent assay (ELISA)

The contents of vascular endothelial growth factor (VEGF) and vascular endothelial growth factor receptor 2 (VEGFR2) in cell supernatant were determined with ELISA kits (R&D, Minneapolis, MN). The absorbance at 450 nm was estimated by microplate reader (Perlong, Beijing, China). Each group was replicated five times.

### Western blot analysis

The total proteins were extracted from cells using RIPA buffer (Beyotime Institute of Biotechnology, China), and the protein concentration was detected with BCA Protein Assay Kit (Dingguo, Beijing, China). The samples per well were separated by 10% SDS-PAGE (Bio-Rad, Hercules, CA) and electrophoretically transferred to PVDF membranes (Millipore, USA). The membranes were incubated overnight at 4°C with primary antibodies (anti-ANGPTL3, anti-Ki67, anti-PCNA, anti-MMP2, anti-MMP9, anti-Integrin-αv, anti-Integrin-β3, anti-αvβ3, and anti-GAPDH), followed by incubation with horseradish peroxidase-labeled secondary antibody (Cell Signaling Technology) for 1 h. The protein bands were exposed using chemiluminescence BeyoECL Plus (Beyotime) and analyzed using Image Software (NIH, Bethesda, MD, USA).

### Statistical analyses

The data were analyzed using SPSS 21.0 software and GraphPad Prism 5 (San Diego, CA, USA). Results are expressed as means ± SD. Comparisons among multiple groups were made with the help of one-way analysis of variance (ANOVA), and Bonferroni post hoc test was also used. It was deemed to be statistically significant when *p* value was less than 0.05.

## Results

### ANGPTL3 is upregulated in CC cells

**Figure 1.**
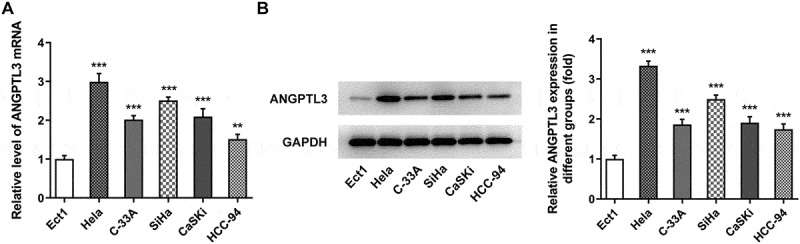
ANGPTL3 is highly expressed in cervical cancer cells. (a) mRNA level of ANGPTL3 in cervical cancer cell lines detected by qRT-PCR. (b) Protein level of ANGPTL3 in cervical cancer cell lines measured by Western blot assay. Results are expressed as mean ± SD. **p < 0.01, ***p < 0.001 versus control.

To disclose the expression of ANGPTL3 in CC, qRT-PCR and Western blot assays were conducted. The results showed that the mRNA level of ANGPTL3 was obviously enhanced in several CC cells relative to that in normal cervical cell line Ect1 (Figure 1a). Consistently, Western blotting results revealed that protein content of ANGPTL3 was notably elevated in CC cells rather than in control normal cervical cells (Figure 1b). Thus, HeLa cell line was selected for the study. These results indicate that the ANGPTL3 expression is associated with the development of CC.

### ANGPTL3 silencing suppresses cell proliferation, invasion and migration of HeLa cells

**Figure 2.**
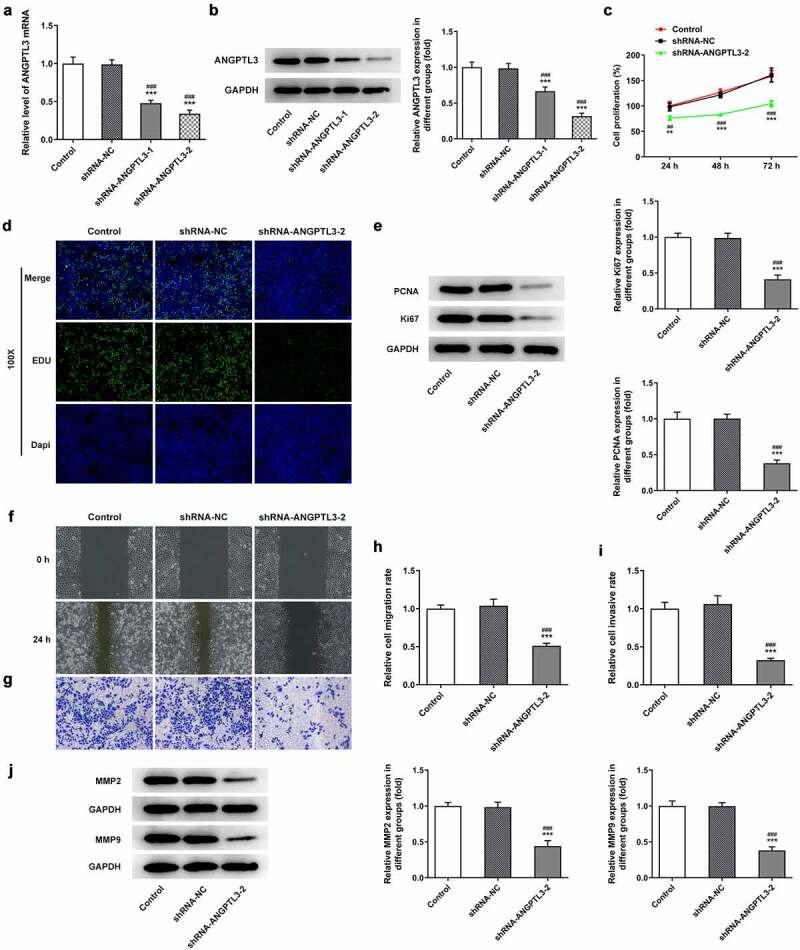
The silencing of ANGPTL3 inhibited the proliferation, invasion and migration of HeLa cells. The mRNA level (a) and protein expression (b) of ANGPTL3 in HeLa cells transfected with or without shRNA-ANGPTL3-1/2. (c) The proliferative capacity of cells was detected by MTT assay at indicated times. (d) EdU staining of transfected HeLa cells. (e) Protein levels of PCNA and Ki67 in HeLa cells measured by Western blot assay. (f) and (h) The migration of cells was identified by wound scratch assay. (g) and (i) Transwell assay appraised cell invasion. (j) Western blot assay measured MMP2 and MMP9 protein levels. Results are expressed as mean ± SD. **p < 0.01, ***p < 0.001 versus control. ^##^*p* < 0.01, ^###^*p* < 0.001 versus shRNA-NC group.

To investigate the biological role of ANGPTL3 in CC cells, we transfected specific shRNA targeting ANGPTL3 to knock down ANGPTL3 expression in HeLa cells. The transfection efficiency was evaluated as displayed in Figure 2(a,b). Based on the results from qRT-PCR and Western blot analysis, shRNA-ANGPTL3-2 was adapted in the subsequent experiments. Subsequently, we examined cell proliferation, migration and invasion after ANGPTL3 was silenced. As presented in Figure 2c, cell proliferation was inhibited by transfection with shRNA-ANGPTL3 in comparison with the shRNA-NC group. In addition, EdU staining showed that the rate of cell proliferation, compared with the control cells, was remarkably decreased in HeLa cells transfected with shRNA-ANGPTL3 (Figure 2d). Similarly, data from Western blotting displayed a prominent decrease in PCNA and Ki67 protein levels in ANGPTL3-silenced cells (Figure 2e). Furthermore, wound healing assay and transwell assay elucidated that the migration and invasion of cells were abrogated by transfection with shRNA-ANGPTL3 (Figure 2(f**–**i)), which is consistent with the result that knockdown of ANGPTL3 distinctly decreased MMP2 and MMP9 protein levels (Figure 2j). The data suggest that downregulation of ANGPTL3 eases the proliferation, invasion and migration of CC cells.

### Interference with ANGPTL3 promotes inhibition of angiogenesis in HeLa cells

**Figure 3.**
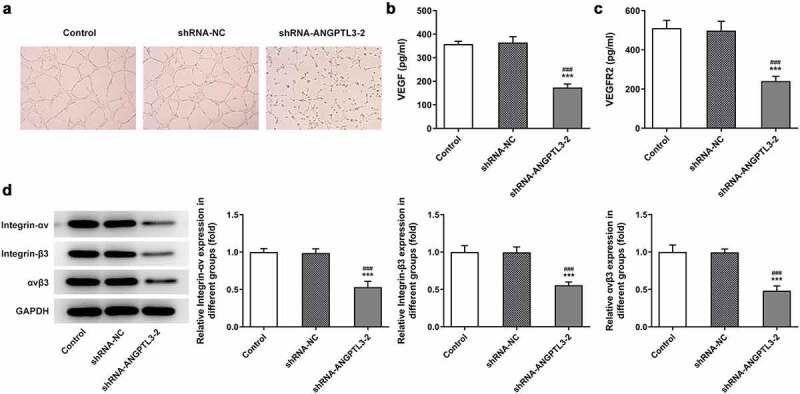
Downregulation of ANGPTL3 inhibits angiogenesis and the expression of integrin αvβ3 in HeLa cells. (a) Angiogenesis in HUVECs cultured in HeLa cell supernatant was estimated by tube formation assay. Levels of VEGF (b) and VEGFR2 (c) in HeLa cells were examined by ELISA. (d) Protein levels of integrin-αv, integrin-β3 and αvβ3 in cells with or without shRNA-ANGPTL3. Results are expressed as mean ± SD. ***p < 0.001 versus control. ^###^*p* < 0.001 versus shRNA-NC group.

To elucidate the effects of ANGPTL3 on angiogenesis of CC cells, we collected cell supernatant from cells transfected with shRNA-ANGPTL3 or shRNA-NC and cultured HUVECs with the cell supernatant. Tube formation assay indicated that silencing of ANGPTL3 notably inhibited the number of tubes and tubulogenesis in HeLa cells (Figure 3a). Additionally, ELISA results indicated that ANGPTL3 insufficiency raised the contents of VEGF and VEGFR2 (Figure 3(b,c)). The data hint the involvement of ANGPTL3 in angiogenesis of CC cells.

### Knockdown of ANGPTL3 represses the expression of integrin αvβ3

We performed Western blot assay to measure the effect of ANGPTL3 silencing on protein expression of integrin αvβ3 in HeLa cells. We found that the levels of integrin-αv and integrin-β3 were significantly decreased after ANGPTL3 was downregulated, as well as αvβ3 protein level (Figure 3d). The results show that ANGPTL3 may regulate integrin αvβ3 expression in CC cells.

### Overexpression of αvβ3 reverses the effect of ANGPTL3 knockdown on proliferation and metastasis of HeLa cells

**Figure 4.**
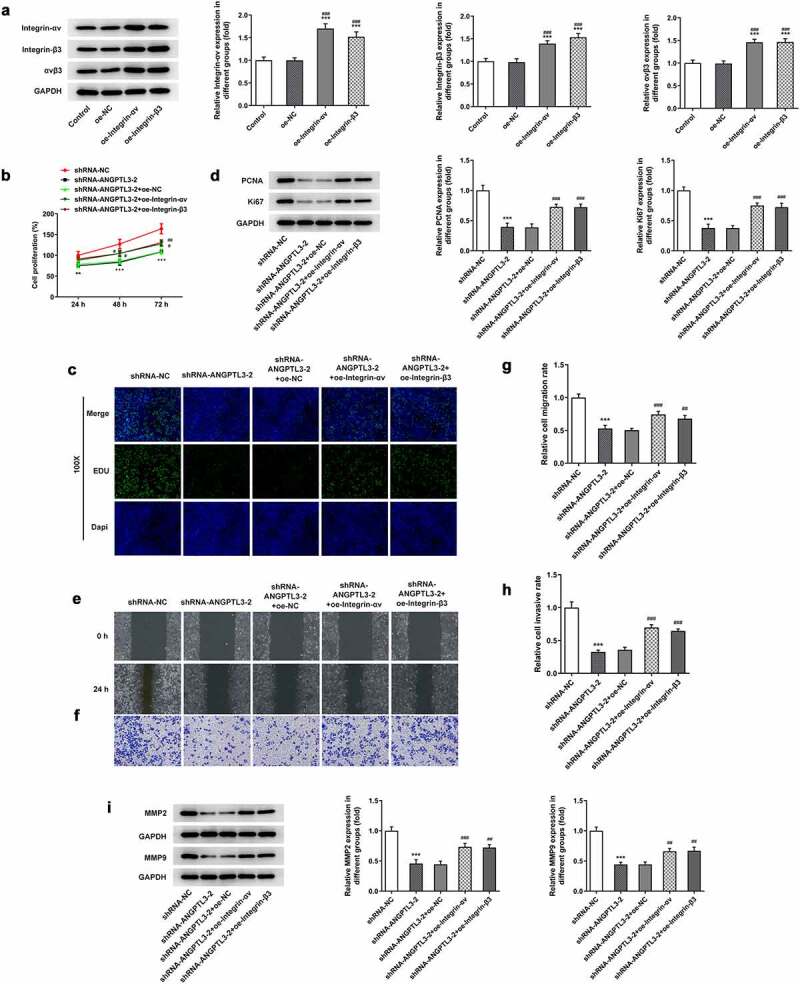
Upregulation of αvβ3 reverses ANGPTL3-mediated HeLa cell proliferation, migration and invasion. (a) Protein levels of integrin-αv, integrin-β3 and αvβ3 after transfection with overexpressed integrin-αv/β3 plasmids. MTT assay (b) and EdU staining (c) were performed to evaluate HeLa cell proliferation. (d) PCNA and Ki67 protein levels were analyzed by Western blot. (e) and (g) Wound scratch assay determined cell migration. (f) and (h) Transwell assay appraised cell invasion. (i) MMP2 and MMP9 protein levels were analyzed by Western blot. Results are expressed as mean ± SD. **p < 0.01, ***p < 0.001 versus shRNA-NC. ^#^*p* < 0.05, ^##^*p* < 0.01, ^###^*p* < 0.001 versus shRNA-ANGPTL3-2+ oe-NC.

To explore the effects of αvβ3 on ANGPTL3-mediated cell proliferation, invasion and migration, we overexpressed αvβ3 in HeLa cells (Figure 4a). MTT assay and EdU staining manifested that the cell proliferative ability was inhibited by interference of ANGPTL3, while overexpression of integrin-αv or integrin-β3 prevented the suppressive role of ANGPTL3 deficiency in cell proliferation (Figure 4(b,c)). Also, the levels of PCNA and Ki67 were observed to be repressed by transfection with ANGPTL3, but upregulation of integrin-αv or integrin-β3 enhanced the decreased protein levels (Figure 4d). Wound healing assay and transwell assay disclosed an increased migratory and invasive rate in cells co-transfected with shRNA-ANGPTL3 and integrin-αv/β3 compared with cells transfected with shRNA-ANGPTL3 (Figure 4(e–h)), which was in line with the Western blot results (Figure 4i). The results indicate that ANGPTL3 silencing undermined cell proliferation and metastasis by at least in part regulating αvβ3 expression.

### Upregulation of αvβ3 reversed the inhibitory effect of ANGPTL3 silencing on angiogenesis of HeLa cells

**Figure 5.**
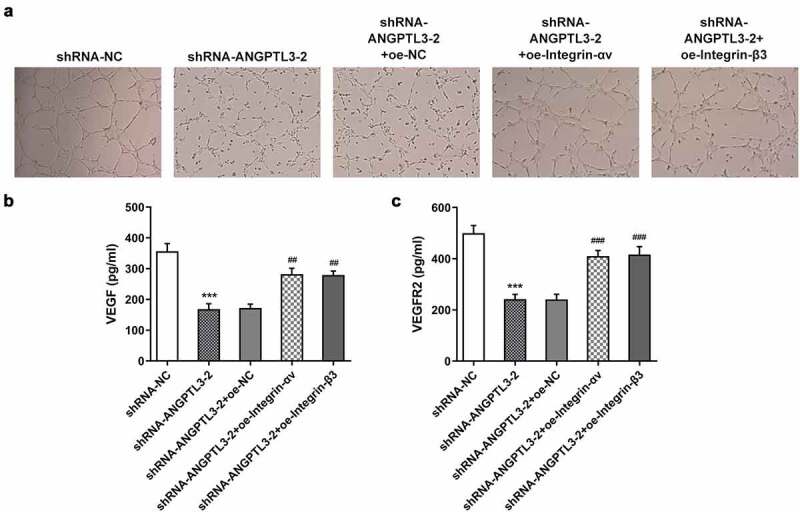
Overexpression of αvβ3 recovers angiogenesis inhibited by ANGPTL3 knockdown in HeLa cells. (a) Angiogenesis in HUVECs cultured in HeLa cell supernatant was estimated by tube formation assay. Levels of VEGF (b) and VEGFR2 (c) in HeLa cells were determined by ELISA. Results are expressed as mean ± SD. ***p < 0.001 versus shRNA-NC. ^##^*p* < 0.01, ^###^*p* < 0.001 versus shRNA-ANGPTL3-2+ oe-NC.

Finally, we examined the role of αvβ3 in angiogenesis of HeLa cells with ANGPTL3 silencing. The results showed that silencing of ANGPTL3 significantly suppressed angiogenesis in HUVECs, whereas transfection with integrin-αv or integrin-β3 rehabilitated the repressed ability of angiogenesis (Figure 5a). Moreover, using ELISA, the levels of VEGF and VEGFR2 were found to be decreased in ANGPTL3-silenced cells but reversed after transfection with integrin-αv or integrin-β3 (Figure 5(b,c)). These data suggest that αvβ3 played a role in the regulatory effect of ANGPTL3 on angiogenesis of HeLa cells.

## Discussion

Cervical cancer (CC) has been a serious threat to women’s health and life for a long time, but its high rate of recurrence and characteristic of metastasis result in defective therapeutic effect and poor prognosis [20,21]. Previous study indicated that upregulation of PTAR could promote cell proliferation, migration and invasion in HeLa cells, which were reversed by PTAR knockdown [22]. Another study demonstrated that RAS p21 protein activator 4 (RASA4) could inhibit the proliferation of cervical cancer cells by inactivating the HIFα signaling pathway [23]. In this study, we revealed that ANGPTL3 was highly expressed in CC cell lines and ANGPTL3 reduction inhibited proliferation, migration and invasion and repressed angiogenesis in HeLa cells. Moreover, we provide evidence supporting the role of integrin αvβ3 in the regulation of ANGPTL3 in CC cells. Overexpression of αvβ3 reversed the influences of ANGPTL3 on physiological and pathological processes of CC cells, suggesting the potential role of ANGPTL3 and αvβ3 as therapeutic targets for CC disease.

It is widely accepted that angiopoietin-like proteins are involved in the development of many types of tumors [24–27]. Previous studies have shown that ANGPTL3 expression was upregulated in several cancer cells or tissues [10,28,29]. In this study, we found that ANGPTL3 was highly expressed in several CCs examined. To explore the functional roles of ANGPTL3 in the progression of CC, the specific shRNA targeting ANGPTL3 was transfected into HeLa cells in which ANGPTL3 expression was highest in CC cells used in this study to knock down the expression of ANGPTL3. The data revealed that the silencing of ANGPTL3 significantly limited the capacities of cell proliferation, migration and invasion. In accordance with our studies, Koyama et al. reported that ANGPTL3 knockdown inhibited cellular proliferation in all shANGPTL3 cells compared with the cells in negative control [9]. In addition, ANGPTL3 was found to be tightly linked with the process of blood vessel formation [30]. Camenisch et al. revealed that ANGPTL3 promoted angiogenesis and functions as a proangiogenic factor using a mouse corneal assay [19]. In our study, angiogenesis in HeLa cells was suppressed after downregulation of ANGPTL3 by observation of tubule formation assay and detection of VEGF and VEGFR2 level. These data demonstrated that ANGPTL3 silencing plays positive roles in inhibition of CC proliferation, metastasis and angiogenesis.

Integrin αvβ3 is one of the most important cell surface molecules that regulates the invasion characteristics of CC cells [31,32]. Recent studies have manifested that ANGPTL3 could regulate endothelial cells via binding to integrin αvβ3 [33,34]. According to data from ENCORI database (http://starbase.sysu.edu.cn), ANGPTL3 is positively correlated with αvβ3 expression in cervical cancer. Our results showed that transfection with shRNA-ANGPTL3 significantly reduced the contents of integrin-αv, integrin-β3 and αvβ3 by Western blot analysis, indicating the regulation of ANGPTL3 in integrin αvβ3 expression.

It has been reported that αvβ3-mediated phosphorylation of FAK and PI3K contributed to invasion and migration stimulated by hypoxic conditions in CC cell lines HeLa and SiHa [35]. Besides, the combination of serum amyloid A1 and integrin αvβ3 increased cell migration and progression in glioblastoma cells [36]. In addition, integrin αvβ3 has been confirmed as a molecular marker to impede tumor-evoked angiogenesis due to its angiogenesis effects [37,38]. Of further interest, we tested the possible relationship between ANGPTL3 and αvβ3 and the role of αvβ3 in participating CC development. We overexpressed αvβ3 in HeLa cells and found that upregulated αvβ3 counteracted the impacts of ANGPTL3 downregulation on the proliferation, metastasis, and angiogenesis in HeLa cells, indicating the involvement of αvβ3 in modulation of ANGPTL3 for biological activities of CC.

## Conclusion

Our study demonstrates that ANGPTL3 can exert tumor-promoting effects in CC cells. Moreover, ANGPTL3 silencing plays a crucial role in inhibiting CC cell growth, metastasis and blood vessel formation via binding to integrin αvβ3. The present study provides a novel fundamental insight into how ANGPTL3 promotes metastasis and angiogenesis in CC. However, there are also some existing limitations. Further, many of the mechanistic observations that are made in vitro in the immortalized cells should be recapitulated in vivo (or in primary isolated cells) to prove that they are causative. In addition, a mechanistic approach on whether the changes observed are merely secondary to ANGPTL3 or αvβ3 or primarily mediated as a result of ANGPTL3-αvβ3 binding inhibition will be explored in our future study.

## Data Availability

The datasets used and/or analyzed during the current study are available from the corresponding author on reasonable request.
